# Inhibition of LRRK2 kinase activity stimulates macroautophagy^☆^

**DOI:** 10.1016/j.bbamcr.2013.07.020

**Published:** 2013-12

**Authors:** Claudia Manzoni, Adamantios Mamais, Sybille Dihanich, Rosella Abeti, Marc P.M. Soutar, Helene Plun-Favreau, Paola Giunti, Sharon A. Tooze, Rina Bandopadhyay, Patrick A. Lewis

**Affiliations:** a Department of Molecular Neuroscience, UCL Institute of Neurology, Queen Square, London, WC1N 3BG, UK; b Reta Lila Weston Institute and Queen Square Brain Bank, UCL Institute of Neurology, 1 Wakefield Street, London, WC1N 1PJ, UK; c London Research Institute, Cancer Research UK, Lincoln's Inn Fields, London, WC2A 3LY, UK; d School of Pharmacy, University of Reading, Whiteknights, Reading, RG6 6AP, UK

**Keywords:** LRRK2, Leucine Rich Repeat Kinase 2, mTOR, Mammalian target of rapamycin, ROC, Ras of Complex Proteins, COR, C-terminal of ROC domain, SDS, Sodium dodecyl sulphate, EDTA, Ethylene di-ammonium tetra acetic acid, DPBS, Dulbecco's phosphate buffered saline, DMSO, Dimethylsulfoxide, LRRK2, Macroautophagy, Parkinson's disease, LC3, p62, WIPI2

## Abstract

Leucine Rich Repeat Kinase 2 (LRRK2) is one of the most important genetic contributors to Parkinson's disease. LRRK2 has been implicated in a number of cellular processes, including macroautophagy. To test whether LRRK2 has a role in regulating autophagy, a specific inhibitor of the kinase activity of LRRK2 was applied to human neuroglioma cells and downstream readouts of autophagy examined. The resulting data demonstrate that inhibition of LRRK2 kinase activity stimulates macroautophagy in the absence of any alteration in the translational targets of mTORC1, suggesting that LRRK2 regulates autophagic vesicle formation independent of canonical mTORC1 signaling. This study represents the first pharmacological dissection of the role LRRK2 plays in the autophagy/lysosomal pathway, emphasizing the importance of this pathway as a marker for LRRK2 physiological function. Moreover it highlights the need to dissect autophagy and lysosomal activities in the context of LRRK2 related pathologies with the final aim of understanding their aetiology and identifying specific targets for disease modifying therapies in patients.

## Highlights

• Inhibiting the kinase activity of LRRK2 induces autophagy
• This induction is independent of an impact on the translational targets of mTORC1
• Inhibition of LRRK2 kinase activity results in a translation dependent increase in p62 levels

## 1 Introduction

Leucine Rich Repeat Kinase 2 (LRRK2) is a multidomain protein of unknown function containing two enzymatic domains, a GTPase (Ras of Complex Proteins, ROC) and a kinase, and several protein/protein interaction domains [1]. LRRK2 has been implicated in a number of cellular processes, including the control of neurite branching, synaptic vesicle recycling, macroautophagy (hereafter referred to as autophagy), protein synthesis through the mammalian target of rapamycin (mTOR) pathway and mitochondrial homeostasis [2]. The physiological function of LRRK2 in the regulation of these processes is, however, unclear.

The central role of this protein in Parkinson's disease (PD) has been highlighted by the discovery of autosomal dominant mutations in *LRRK2* causing familial Parkinson's disease and the subsequent identification of the *LRRK2* locus as a risk factor for sporadic disease [3,4]. A key question regarding the role of autosomal dominant coding change mutations in PD is what the cellular consequences of these mutations are, and how they lead to disease [2]. Penetrant coding mutations are found exclusively in the enzymatic core of LRRK2 — the ROC/COR/kinase triptych [4], leading to a number of studies examining the impact of mutations on the enzymatic activities of this protein. The G2019S mutation, the most common disease linked variant in LRRK2, has been consistently associated with increased kinase activity, and mutations in the ROC and COR domains display reduced GTPase activity [5–9]. However, thus far no biochemical phenotype has been consistently linked to mutations in all three of these domains. The only reported cellular phenotype that consistently correlates with penetrant mutations is cytotoxicity, which is dependent upon kinase activity [10–12].

A number of recent reports have suggested a role for LRRK2 in the autophagy/lysosomal pathway [13–21]. Data from a range of cell lines and patient derived cells have revealed alterations in key markers of autophagy in the presence of mutations in LRRK2, although the precise point in the pathway that links LRRK2 to this process has not been identified [13,14,18–20]. The relationship between LRRK2 and autophagy has been further highlighted by studies in animal models lacking LRRK2 or expressing a mutant form of the protein [15,16,21]. Knockdown studies support a complicated link between LRRK2 and the induction/regulation of autophagy, in particular the demonstration that loss of LRRK2 results in biphasic changes in autophagy over the course of mouse development [21]. Data from fly models of LRRK2 dysfunction have suggested that LRRK2 may function in the mTOR pathway, implicating LRRK2 in a pathway with an important role in regulating autophagy, although these data have proved controversial [22,23]. Intriguingly, LRRK2 has also been identified as a risk factor in a number of human diseases characterized by a strong pathogenic link to autophagy (in addition to PD): Crohn's disease, cancer and leprosy [24–26]. A key research challenge in LRRK2 biology is, therefore, to elucidate the precise role of this protein in autophagy.

To clarify the role of LRRK2 in the regulation of autophagy, this study takes advantage of recently described inhibitors of LRRK2 kinase activity [27,28] to test whether the kinase activity of endogenous LRRK2 is important for this pathway at a cellular level, and to delineate the point at which LRRK2 intervenes in autophagy.

## 2 Materials and methods

### 2.1 Inhibitors

The LRRK2-in1 and the CZC-25146 compounds were purchased from the Department of Biochemistry, University of Dundee, UK. GSK 2578215A was purchased from Tocris Bioscience. Bafilomycin A1 (B1793-2UG) and cyclohexamide (01810-1G) were purchased from Sigma-Aldrich.

### 2.2 Antibodies

Antibodies used were as follows: rabbit LC3 antibody (NB100-2220, Novus Biologicals); mouse LC3 antibody (5F10, Nanotools), LRRK2 antibodies (N138/6, NeuroMab and 3514-1, Epitomics); total S6 antibody (2317, Cell Signalling); phospho Ser235/236S6 antibody (2211S, Cell Signalling); total P70S6K antibody (sc-8418, Santa Cruz); phospho Thr389 P70S6K (sc-11759, Santa Cruz); total 4EBP1 (81149, Santa Cruz); phospho Ser65 4EBP1 (9451S, Cell Signaling); mouse p62 antibody (610833, BD Transduction Labs); rabbit p62 antibody (BML-PW9860-0025, Enzo Life Sciences); mouse WIPI2 antibody (kindly supplied by Prof. S. Tooze) and mouse β-actin antibody (A1978, Sigma Aldrich). LRRK2 phosphorylation was assessed using rabbit phospho Ser935-LRRK2 (5099-1, Epitomics).

### 2.3 Cell culture, cell treatments

Cell lines were grown in DMEM containing 10% FCS, with the exception of the mTOR stimulation experiment as described below. Human neuroglioma H4 cells (ATCC number HTB-148), human neuroblastoma SHSY5Y (ATCC number CRL-2266) or Human Embryonic Kidney (HEK) cells (ATCC number CRL-1573) were seeded at a concentration of 2 × 10^5^ cell/ml in 6 wells plates (2 ml for each well). After 6 hours from plating, cells were treated with LRRK2 inhibitors LRRK2-in1, CZC-25146 and GSK 2578215A. The concentrations of inhibitors, as used during the treatment, are reported in every experiment shown in the text. All compounds were dissolved in DMSO. For each experiment, DMSO vehicle controls were added. Cells were incubated overnight with LRRK2 kinase inhibitors and fresh treatment was replaced the following morning for 2.5 hours before cell lysis.

Cells were then washed once in Dulbecco's phosphate buffered saline (DPBS) and collected in a lysis buffer containing: 0.5% Triton X-100, 2 mM ethylene di-ammonium tetra acetic acid (EDTA), 150 mM NaCl, 0.5% sodium deoxycholate, 0.1% sodium dodecyl sulphate (SDS), protease inhibitors (cOmplete, protease inhibitor cocktail, Roche) and phosphatase inhibitors (Halt phosphatase inhibitor cocktail, Pierce) in 50 mM TRIS-HCl pH 7.5.

For mTOR pathway experiments, cells were seeded as described above; mTOR inhibition was achieved by overnight (16 hours) serum deprivation followed by substitution of the growing medium with Earle’s balanced salts solution for 2 hours. Re-activation of the mTOR pathway was obtained after starvation by feeding cells with MEM non-essential amino acid supplement added directly to the Earle's solution for 30 minutes. Non-starved, starved and amino acid fed cells were then washed once in DPBS and collected in lysis buffer.

### 2.4 Primary astrocyte preparation

Primary astrocytes from cortex were isolated from 3 days old rats as previously described [30]. The tissue was mechanically dissociated and trypsinized; the obtained cell pellet was plated in high glucose DMEM containing 10% FBS. After 5 days, cell medium was changed to 20% FBS and then replaced every 5 days. At 15 days microglia were detached by shaking of the flasks at 200 rpm for 2 hours. The remaining astrocytes were washed in PBS, trypsinized and seeded 1:2 in new flasks and kept in culture by splitting 1:2 when at confluency. Astrocytes were used for experiments after 21 or 27 days in culture. They were seeded 1 × 10^6^ in 6 wells plates and treated with LRRK2-in1 as described for H4 cells or plated 2.5 × 10^5^ in 24 wells plates containing glass coverslips coated in 1 mg/ml poly-d-lysine for cell imaging. Purity was accessed by immunostaining with anti-GFAP antibody (1:1000, Abcam) and DyLight 594 Labeled GSL I-isolectin B4 (1:50, Vector Laboratories).

### 2.5 Immunoblotting

Cell lysates were frozen immediately upon collection; following thawing, they were clarified by centrifugation at 10,000*g* for 5 minutes at 4 °C prior to use. Lysate protein concentrations were assessed by BCA assay (BCA Protein Assay Kit, Pierce) and samples containing 10 μg of proteins were prepared for SDS-PAGE with the addition of NuPAGE sample buffer (Invitrogen), and denatured for 10 minutes at 70 °C. Electrophoresis was performed using NuPAGE, Novex precasted Bis-Tris 4–12% gels (Invitrogen), according to the manufacturer's instructions. After electrophoresis, proteins were transferred to 0.45 μm PVDF membranes (IPVH00010, Immobilon Millipore) for 2 hours. Proteins were identified by the appropriate primary and secondary HRP antibodies and visualized using Enhanced Chemiluminescence (ECL) and X-Ray films (Super RX, medical X-Ray film, Fujifilm). Films were acquired as images in jpg format using an EPSON Perfection 4870 photo scanner and processed by the ImageJ software (http://rsbweb.nih.gov/ij/) to quantify area and total intensity of each single band. Statistical analyses were performed by Prism software (GraphPad) as described in the text.

### 2.6 Generation of shRNA scramble control/LRRK2 knockdown (KD) stable H4 cell line

H4 cells were transfected with 2 μg LRRK2 shRNA or scramble shRNA (V3LHS-644167, Thermo Fisher Scientific) using Effectene (Qiagen) transfection reagent according to the manufacturer's instructions. ShRNA vectors contain a puromycin resistance gene therefore 48 hours post transfection cells were treated with 2 μg/ml puromycin supplemented DMEM. Media were changed every 2 days (removal of dead cells) for 2 weeks in order to select for puromycin-resistant cells containing shRNA.

### 2.7 Neutral red staining

Cells were treated with DMSO, LRRK2-in1 (1 μM over night) or with the autophagy inhibitor bafilomycin (40 nM, over night); at the end of the treatment, the cell culture medium was supplemented with a solution of 3-amino-7-dimethylamino-2-methyl-phenazine hydrochloride (Neutral red, Sigma Aldrich) with a final concentration of 80 ng/ml, for 30 minutes [29]. Cells were washed twice with DPBS and dissolved in a destaining solution composed of 50% ethanol, 49% deionized water, 1% glacial acetic acid and the absorbance was recorded by the use of a multiwell plate reader at the wavelength of 540 nm. For every 96 well plates used in the assay, a replicate of 2 wells per single column was used to determine protein concentration (BCA assay). Data were expressed as absorbance at 540 nm normalized to the absorbance recorded for the BCA assay for every single column within the plate. The final results in the graph were expressed as percentage of Neutral red staining in comparison with untreated controls.

### 2.8 Cytotoxicity

Cells were treated with LRRK2 kinase inhibitors as described above; at the end of the treatment, the cell culture medium was added of 3-(4,5-dimethylthiazol-2-yl)-2,5-diphenyltetrazolium bromide (MTT, Sigma Aldrich) to the final concentration of 500 μg/ml for 3 hours. Cell medium was then discarded and the formazan crystals accumulated within the energetically active cells were dissolved in 100% DMSO and the absorbance measured using a multiwell plate reader at 570 nm. The results were reported as percentage of cell viability after treatment in comparison with DMSO treated, control cells.

### 2.9 Immunocytochemistry

Cells were seeded onto a sterilized coverslip in 24 wells plates at the concentration of 2 × 10^5^ cell/ml (0.5 ml each well). Post treatments, cells were washed twice in DPBS and fixed a room temperature for 15 minutes in a solution of 4% paraformaldehyde in DPBS or in ice cold methanol at 4 °C (only for 5F10, anti-LC3 antibody). Cells were washed three times in DPBS, blocked and permeabilized at room temperature for 30 minutes by using a solution of 15% normal goat serum (S1000, Vector) and 0.1% Triton X-100 in DPBS. After washing, cells were incubated overnight at 4 °C with the primary antibody. Anti-mouse, secondary antibody (Alexa Fluor, emission at 568 nm) or anti-rabbit, secondary antibody (Alexa Fluor, emission at 488 nm) were used to reveal the primary antibody staining and nuclei were labeled with Hoechst 33342. Coverslips were sealed with Fluoromount G mounting medium (Southern Biotech). Images were acquired with a Leica CTR 6000 fluorescence microscope, and processed by the LAS AF Lite software or with a Zeiss LSM 710 confocal microscope and processed by the Zen 2009 software.

Cells containing WIPI2 or p62 puncta were manually counted by a blinded operator using the acquired images and the cell counter plugin tool from ImageJ software. Graphs and statistical analyses were performed using Prism software.

For p62 puncta, images were acquired at 40 × magnification (DMSO treated cells: 3 different fields in 6 independent slides; LRRK2-in1 treated cells 1 μM: 3 different fields in 6 independent slides; LRRK2-in1 treated cells 5 μM: 3 different fields in 2 independent slides; cells under starvation: 3 different fields in 4 independent slides; each field contained an average of 130 cells. Wild-type, scrambled and LRRK2 KD H4 cells: 3 different field in 2 independent slides; an average of 305 cells was analyzed for each condition).

For WIPI2 puncta, images were acquired at 63 × magnification (DMSO treated cells: 9 different slides with an average number of 113.6 total cells each; LRRK2-in1 treated cells 1 μM: 9 different slides with an average number of 137.8 cells each; LRRK2-in1 treated cells 5 μM: 2 different slides with an average number of 88.5 total cells each; cells under starvation: 5 different slides with an average number of 146.2 cells each).

Colocalization was determined by the use of the Volocity software. colocalization was calculated following the instructions reported in the Perkin-Elmer website: http://www.perkinelmer.co.uk/pages/020/cellularimaging/training/theorycolocalizationanalysis.xhtml http://www.perkinelmer.co.uk/pages/020/cellularimaging/training/performingcolocalizationanalysisvolocity.xhtml.

After threshold correction for the background, Pearson's correlation coefficient (perfect correlation = 1; no correlation = 0; perfect inverted correlation = − 1) is indicated as PCC; Mander's colocalization coefficients (i.e. showing the overlapping between the channels) are indicated as Mgreen and Mred.

### 2.10 Z-stack movies

Z-stacks were acquired using a Zeiss LSM 710 confocal microscope using the Zen 2009 imaging software (Zeiss). The first frame was fixed by the operator as the first zoom level in which the green signal was visible. The last frame was then selected by unzooming until the green fluorescence disappeared. The thickness of the slices within the total volume between the first and the last frame was selected according to the software suggestions. 3D movies generated by the Zen software based on the acquired z-stacks were selected for speed, rotation angle, zoom and number of frames and were exported as avi files. Avi movies and correspondent freeze-frames were composed in a storyboard by the use of the Windows Live Movie Maker program and finally saved as wmv files.

## 3 Results

**Supplementary Fig. S1:**
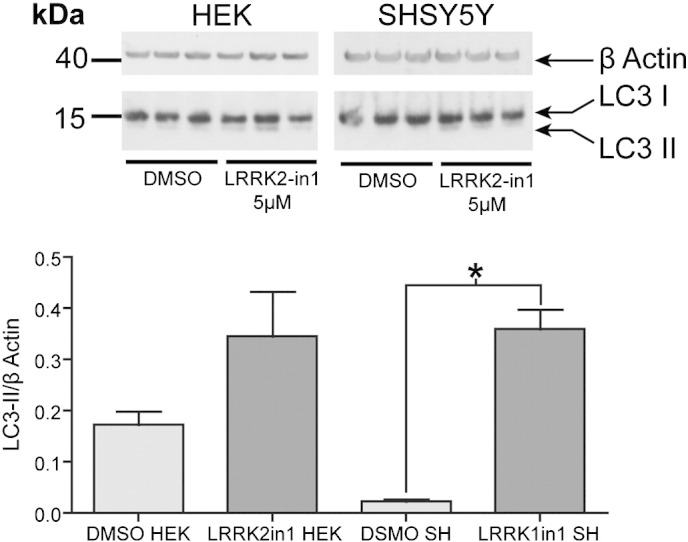
HEK and SHSY5Y have been treated over night with 5 μM LRRK2-in1. As described for H4 cells, an increase in the LC3-II band was detected even if at a lesser extent. Quantification revealed a non-significant increase in LC3-II after inhibitor treatment in comparison with controls in the case of HEK cells, and a statistically significant increase in SHSY5Y cells.

**Fig. 1:**
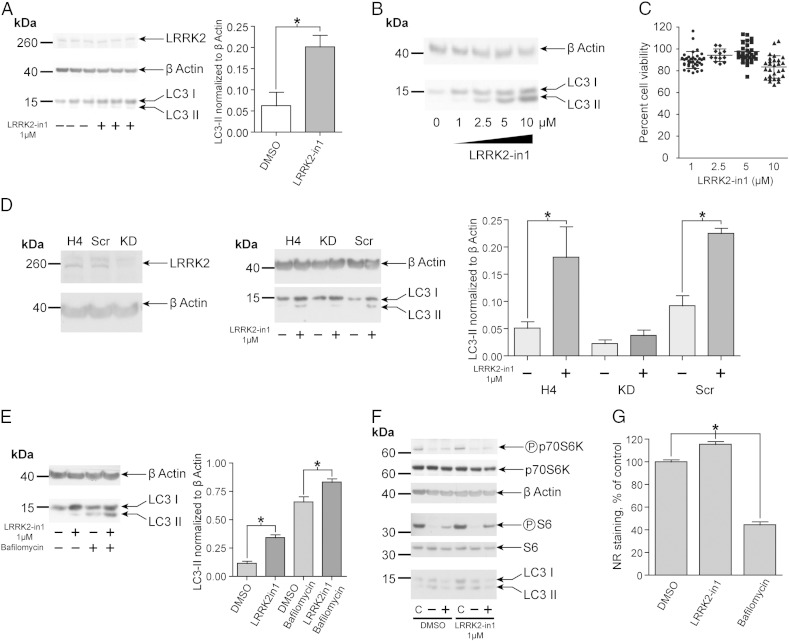
Inhibition of LRRK2 alters the autophagy/lysosomal pathway in H4 neuroglioma cells. A) LC3-II levels increase upon LRRK2-in1 inhibitor treatment (1 μM, overnight treatment; quantification from 3 independent replicates, the plot shows mean and SD, * indicates significance (*p* < 0.05)). B) Dose dependent increase in LC3-II upon overnight treatment with LRRK2-in1. C) MTT assay showing no alteration of cell viability upon over night treatment with LRRK2-in1 from 1 to 5 μM. A small toxic effect appeared with the higher dose (10 μM). The plot shows mean and SD, * indicates significance (*p* < 0.05). D) LRRK2 knockdown cells display reduced response to LRRK2-in1. LRRK2 protein levels are decreased in shRNA stable line compared to wild type cells (right panel), and knockdown of LRRK2 reduces response to 1 μM LRRK2-in1 treatment compared to wild type or scrambled shRNA cells (left panel). E) Western blot analysis of H4 cells treated with DMSO and LRRK2-in1 (5 μM, 2.5 hours treatment) in the presence and absence of 40 nM bafilomycin added at the same time as the inhibitor. Quantification of three replicates is shown in the right hand panel, the plot shows mean and SD, * indicates significance (*p* < 0.05). F) LRRK2-in1 increases LC3-II levels independent of mTORC1 activity. P70S6K and phoshoThr389-P70S6K; S6 and phosphoSer235/236-S6 levels are shown in control, starvation and amino-acid stimulated conditions. LRRK2-in1 (1 μM over night) treatment does not alter phosphorylation of P70S6K and S6 in conditions that show increased levels of LC3-II. G) Neutral red staining. The plot shows mean and SEM. * indicates significance (*p* < 0.05).

Recent data indicate LRRK2 is expressed in glial cells and that it has a key, functional role in orchestrating the inflammatory response of this cell population [31,32]. A number of reports have highlighted dynamic expression of LRRK2 in a range of immune cells; thus leading to the hypothesis that LRRK2 may drive a function related with the innate immune response [33,34]. Based upon these investigations, the human neuroglioma cell line H4 was selected as a model system to investigate endogenous LRRK2 function in non-neuronal populations. Using conversion of LC3-I to LC3-II by conjugation to phosphatidylethanolamine as a marker for alterations in autophagy [35], the cellular response of H4 cells in the presence of a specific inhibitor of LRRK2 kinase function, LRRK2-in1 [28], was examined. Overnight treatment of H4 cells with LRRK2-in1 resulted in a significant increase in the detectable levels of LC3-II compared to vehicle (DMSO) treated cells (Fig. 1A). The increase in LC3-II accumulation followed a dose response trend (Fig. 1B) and was not associated with cytotoxicity (as measured by MTT assay) in the concentration range of 1–5 μM (Fig. 1C). A small toxic effect was recorded with 10 μM inhibitor; for this reason the maximum concentration of LRRK2-in1 used for all subsequent experiments was 5 μM. A similar response to LRRK2 kinase inhibition was observed in SHSY-5Y and HEK293T cells (Supplemental Fig. S1).

**Supplementary Fig. S2:**
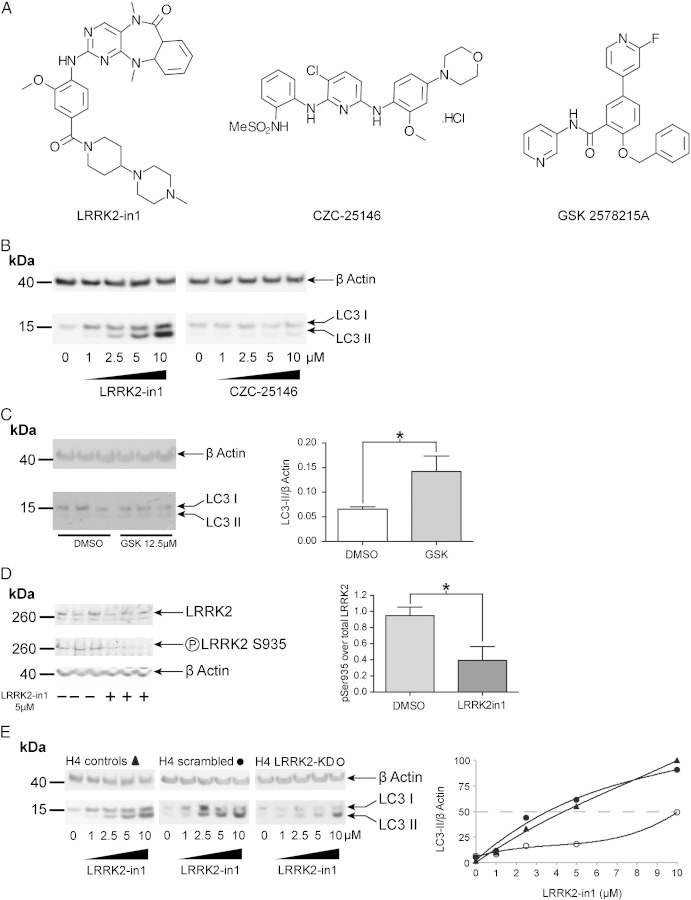
A) Inhibitors used in this study, showing distinct structural properties. B) LRRK2-in1 and CZC-25146 dose response. H4 cells have been treated overnight with LRRK2-in1 or CZC-25146 at 1, 2.5, 5, 10 μM. Cell lysates have been analyzed in Western blot. With both inhibitors an accumulation of LC3-II has been reported in a dose dependent manner, however LRRK2-in1 was more potent than CZC-25146 to promote LC3 conversion. C) Treatment of H4 cells with 12.5 μM GSK 2578215A results in an in increase in LC3-II as seen with LRRK2-in1 and CZC-25146. The plot shows mean and SEM. * indicates significance (*p* < 0.05). D) Treatment of H4 cells with LRRK2-in1 results in loss of phosphorylation on S935 as measured by immunoblot. The plot shows mean and SEM. *significance (*p* < 0.05). E) H4 native, LRRK2 knockdown and scrambled cells, have been treated overnight with LRRK2-in1 at 1, 2.5, 5, 10 μM. Cell lysates have been analyzed in Western blot. A dose dependent accumulation of LC3-II has been observed for the three lines however at a lesser extent in LRRK2 knockdown cells. The 10 μM dose in native H4 was found to be the condition for the maximum accumulation of LC3-II. In consequence, this data point was selected as 100% and all the other data were normalized accordingly. After normalization, native H4 cells and scrambled cell showed the same trend in accumulation of LC3 during LRRK2-in1 treatment, while H4 knockdown cells showed a reduced response to the inhibitor and the calculated EC50 for LC3-II accumulation was reduced by an half and shifted from 5 μM to 10 μM.

To determine if the increase in LC3-II was due to inhibition of LRRK2 kinase activity or to an off target impact of LRRK2-in1, a dose response with a structurally distinct inhibitor of LRRK2 kinase activity, CZC-25146 (Supplemental Fig. S2A), was carried out [27]. This resulted in a similar increase in LC3-II, albeit at higher concentrations than LRRK2-in1 (Supplemental Fig. S2B). A similar response was seen with an additional LRRK2 kinase inhibitor, GSK 2578215A (Supplemental Fig. S2C) [36]. To assess whether treatment of H4 cells with LRRK2-in1 results in inhibition of LRRK2 kinase activity *ex vivo*, analysis of phosphorylation of LRRK2 at residue S935, which can be correlated with LRRK2 kinase activity despite not being a direct autophosphorylation event [28], was carried out. This revealed the expected decrease in phosphorylation in the presence of LRRK2-in1 (Supplemental Fig. S2D).

An H4 LRRK2 stable knockdown line (generated using a shRNA construct, resulting in a ≈ 60% decrease in LRRK2 expression) was then used to test whether the increase in LC3-II upon LRRK2-in1 treatment was a LRRK2 dependent phenomenon, and not an off target effect of the inhibitor. Upon treatment with LRRK2-in1, the LRRK2 knockdown cells displayed a significantly reduced response compared to wild type H4 cells and to scrambled shRNA cells (Fig. 1D), with a concomitant reduction in the response of cells over a range of doses (Supplemental Fig. S2E).

To investigate whether the alteration in LC3-II levels upon inhibition of LRRK2 was due to an induction of autophagy (that is, an increase in the production of LC3-II) or to an alteration in the recycling of LC3-II, the response of H4 cells to LRRK2-in1 was assessed in the presence of bafilomycin, an inhibitor of lysosomal acidification [26] (Fig. 1E). As expected, LC3-II levels were increased in both DMSO and LRRK2-in1 treated cells in the presence of bafilomycin, with the LRRK2-in1 plus bafilomycin treated cells displaying a significantly higher level of LC3-II compared to the LRRK2-in1 only and the DMSO plus bafilomycin treated cells. This suggests that the increase in LC3-II levels in LRRK2-in1 treated cells is due to an increase in production rather than the decreased recycling of the lipid conjugated form of LC3. To examine if LRRK2 kinase inhibition impacts on autophagic recycling using an alternative approach, the presence of low pH vesicles was assessed using neutral red, a dye that is specifically retained by acidic vesicles [29]. As expected, bafilomycin markedly decreased the staining with this dye (Fig. 1F). In contrast, neutral red staining in LRRK2-in1 treated cells was statistically indistinguishable from DMSO treated cells. This supports a role for LRRK2 kinase activity in autophagocytosis independent of vesicle acidification.

**Supplementary Fig. S3:**
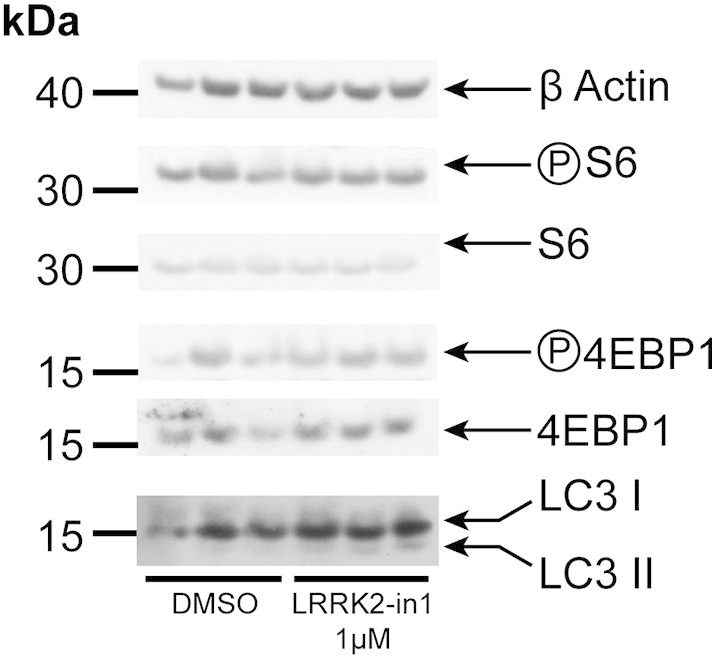
H4 cells treated with LRRK2-in1 have been analyzed for the phosphorylation of 4EBP1 on Ser65. No alterations in the phosphorylation state were detected during inhibitor treatment able to induce LC3-II accumulation (1 μM, over night).

Previous studies have suggested that LRRK2 may operate in the mTOR pathway regulating protein synthesis through the phosphorylation of 4EBP1 [25], although whether this is a physiological interaction is unclear [23,37]. To determine if the alteration in LC3-II levels upon LRRK2 inhibition is due to upstream alterations in the mTOR pathway the phosphorylation of p70S6K, S6 and 4EBP1 (downstream translational inhibition targets of mTORC1) was examined in the presence of LRRK2-in1. To modulate the mTOR pathway, cells were starved of amino acids (inhibiting mTORC1), and then re-exposed to amino acids to re-active the pathway. No reduction in the level of phosphorylation of target proteins was observed in the presence of LRRK2 kinase inhibitors (Fig. 1F, Supplemental Fig. S3), suggesting that, at the endogenous level and in this model system, LRRK2 acts independently of canonical mTORC1 signaling to induce autophagy.

**Fig. 2:**
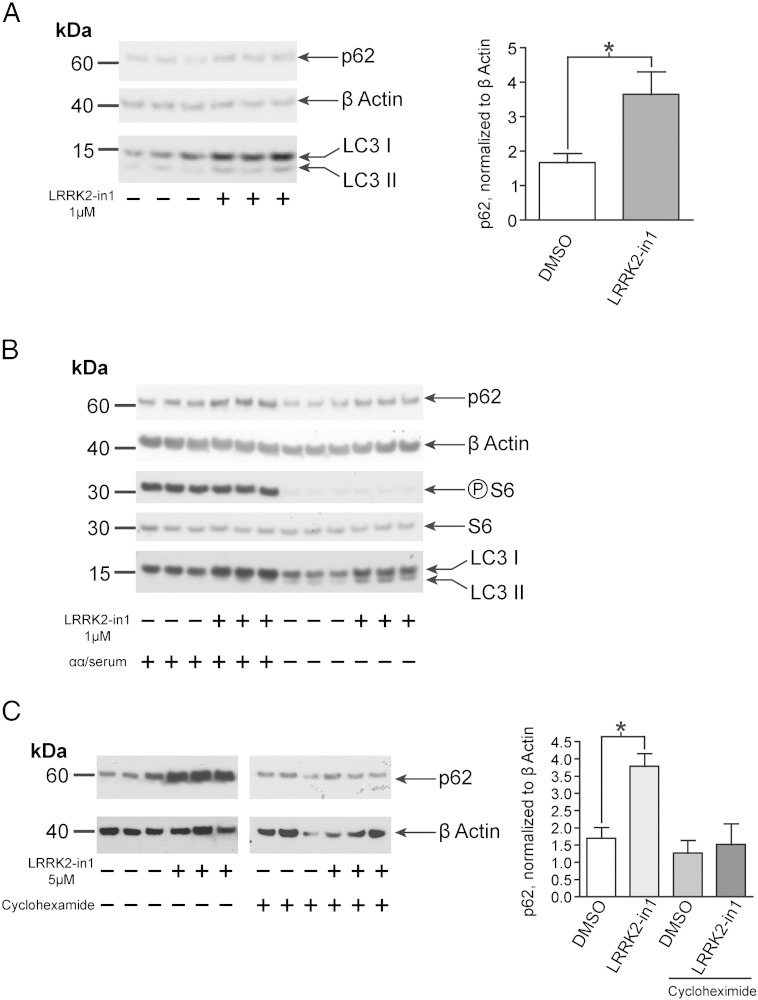
The impact of LRRK2-in1 on p62 levels A) p62 levels increase upon overnight treatment with LRRK2-in1 (1 μM). The plot shows mean and SD, * indicates significance (*p* < 0.05) B) Analysis of p62 levels under control and starvation conditions. Reduced levels of total p62 under starvation conditions for both DMSO and LRRK2-in1 treated cells are detected, however the treatment with LRRK2-in1 (1 μM, over night) is able to impact onto total p62 levels even under starvation. C) Impact of LRRK2-in1 upon p62 in the presence of cyclohexamide. H4 cells treated with DMSO and LRRK2-in1 (5 μM, overnight treatment) in the presence and absence of 50 μg/ml cyclohexamide. The plot show mean and SD, statistical analysis was by ANOVA. * indicates significance (*p* < 0.05).

To examine autophagic clearance, p62 levels were examined in the presence and absence of LRRK2-in1. This revealed a surprising increase in total levels of p62 upon treatment with LRRK2-in1 (Fig. 2A). During starvation (stimulating autophagy through inhibition of mTORC1) an expected decrease in the total level of p62 was shown for both DMSO and LRRK2-in1 treated cells, however the level of p62 still remained higher in starved cells treated with the LRRK2 kinase inhibitor in comparison with starved controls (Fig. 2B). This increase in total p62 levels was not visible upon a shorter treatment with LRRK2-in1 (data not shown), it appeared only after an overnight cell exposure to the compound. Based upon a previous report by Gomez-Suaga and co-workers [17], who observed a translation-dependent increase in p62 levels upon manipulation of LRRK2, LRRK2-in1 treatment was carried out in the presence of cyclohexamide. This blocked the previously observed increase in p62, suggesting this phenomenon is driven by increased synthesis of p62 rather than decreased turnover (Fig. 2C). More experiments are required to verify if this is a direct effect due to LRRK2 kinase inhibition or whether it is an off target effect of the LRRK2-in1 compound.

**Supplementary Fig. S4:**
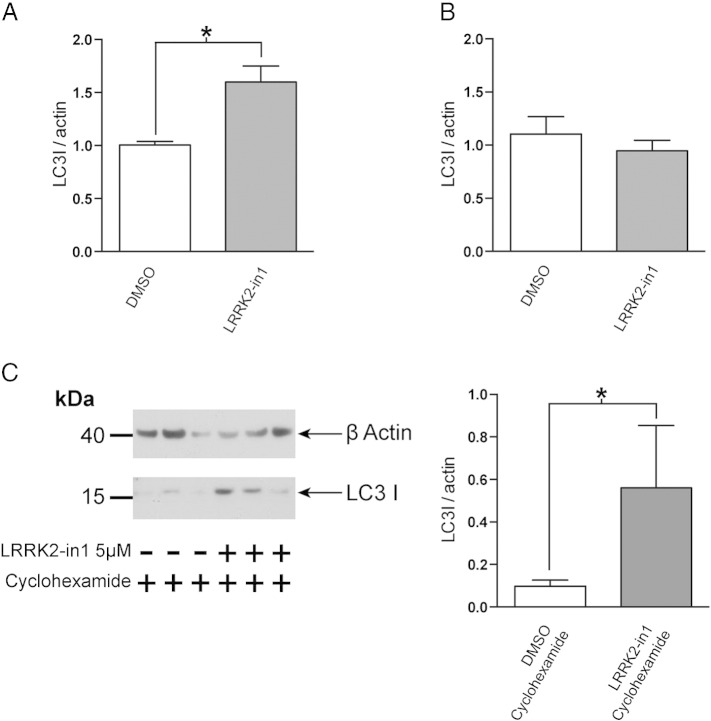
A) An increase in LC3-I is observed in H4 cells after overnight inhibition of LRRK2 kinase activity by LRRK2-in1, 1 μM. B) This increase is not observed after 2.3 hour treatment with LRRK2-in1, 1 μM. C) The increase in LC3-I upon LRRK2-in1 treatment is not dependent upon translation. Treatment of cells with LRRK2-in1 (5 μM overnight) in the presence of cyclohexamide resulted in an increase in LC3-I, in contrast to results gained with p62 (see Fig. 2). The plot shows mean and SEM. *Significance (*p* < 0.05) by paired *t*-test.

It is noteworthy that, in parallel to the increased levels of LC3-II, an increase in LC3-I levels is also observed (for example Supplemental Fig. S2B). In H4 cells this occurs only after 24 hours of LRRK2 kinase inhibition, and is not observed after 2.5 hours of inhibition (Supplemental Fig. S4), suggesting that this is a consequence of the increased autophagic flux rather than driving this phenomenon. In contrast to p62, inhibition of translation with cyclohexamide, while decreasing LC3 levels globally, does not alter the increase in LC3-I levels in response to LRRK2 kinase inhibition (Supplemental Fig. S4). This suggests that this phenomenon is not due to an increase in the translation of LC3, instead being a result of the increase in autophagic flux upon inhibition of LRRK2 kinase activity.

**Fig. 3:**
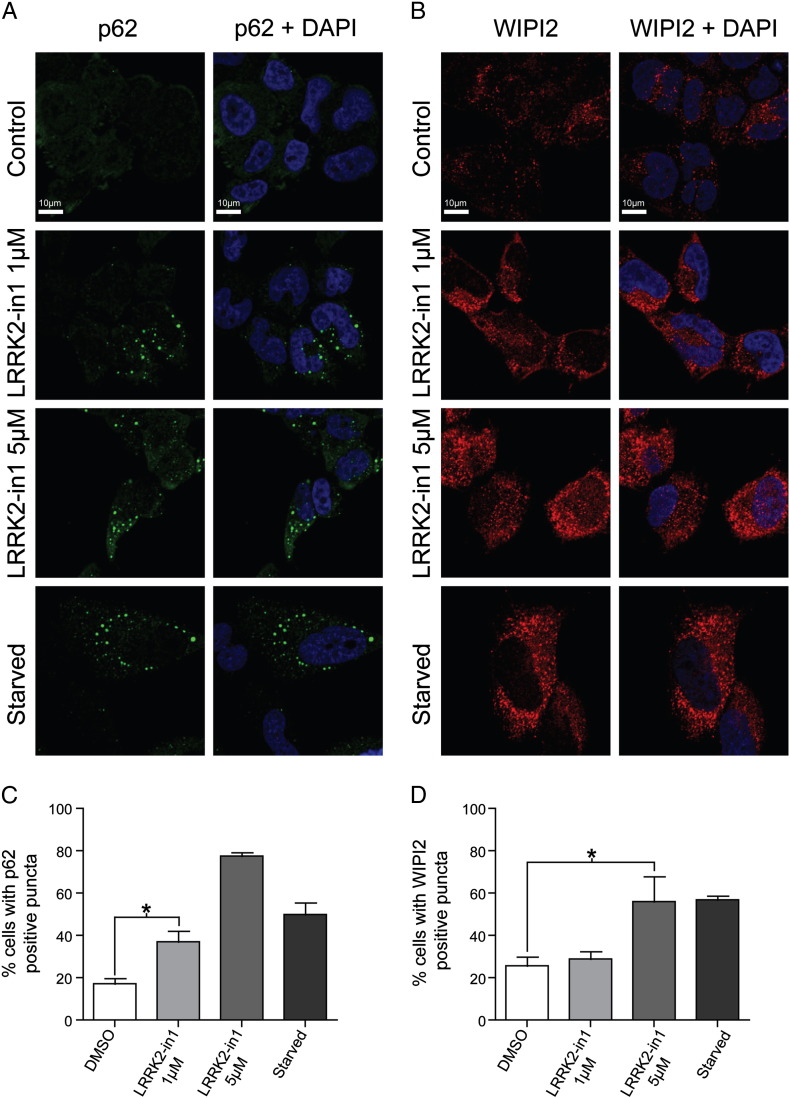
Immunocytochemical analysis of control, LRRK2-in1 (1 and 5 μM) treated and starved H4 cells. A) p62 (green), B) WIPI2 (red) and nuclear staining in blue. Scale bar represents 10 μm. Mean and SD are displayed for both plots, statistical analysis was by ANOVA. *indicates significance (*p* < 0.05).

**Fig. 4:**
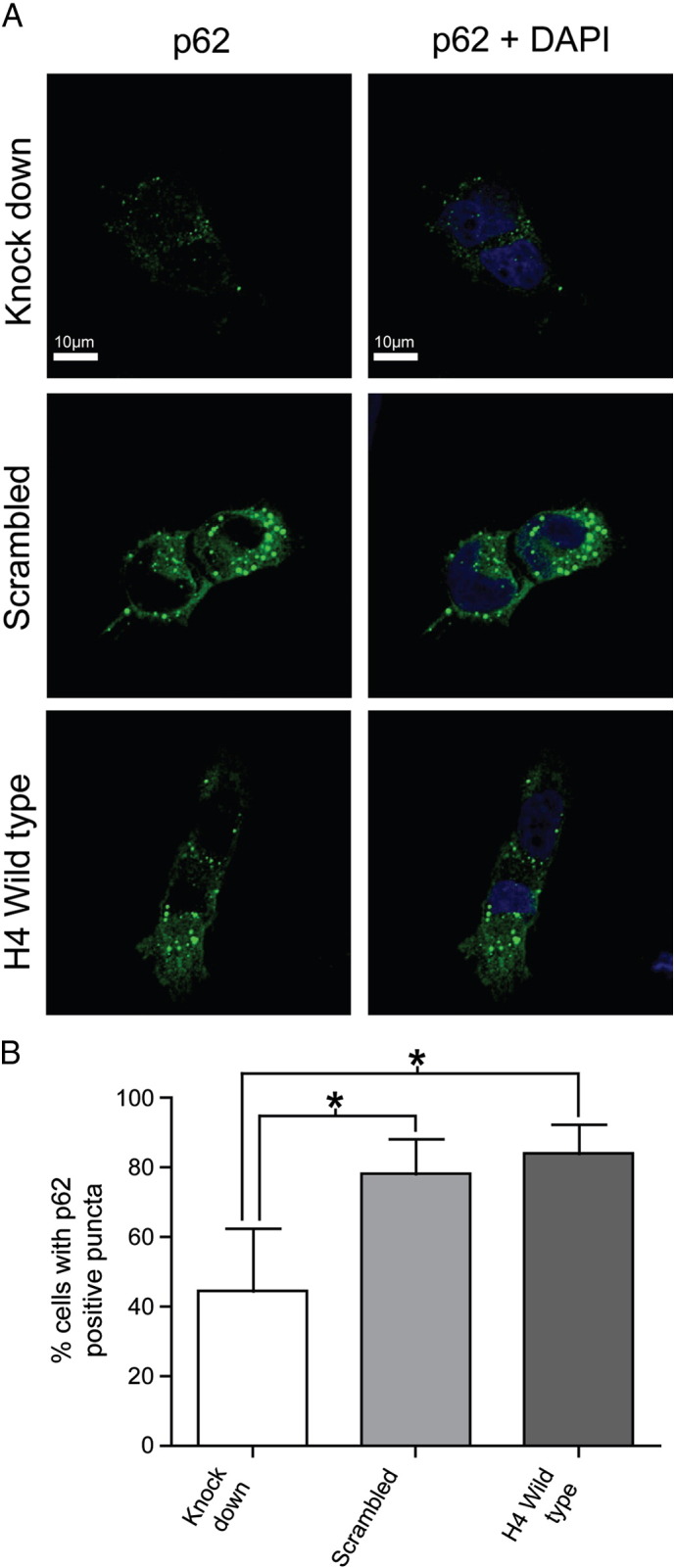
Immunocytochemical analysis of control, scrambled and LRRK2 knock down H4 cells after 5 μM LRRK2-in1 over night treatment. p62 (green) and nuclear staining in blue. Mean and SD are displayed in the plot, statistical analysis was by ANOVA. *indicates significance (*p* < 0.05).

To confirm the inhibition of LRRK2 kinase activity results in a change in the autophagy flux similar to the effect of starvation, the formation of autophagosomes was assessed by immunocytochemistry. Treatment with LRRK2-in1 at two different concentrations resulted in the formation of p62 clusters and WIPI2 positive puncta, similar to that expected and observed in cells in which autophagy has been stimulated by starvation [38]. The percentage of cells containing p62 clusters or WIPI2 positive puncta was significantly increased upon inhibitor treatment (Fig. 3) but not in cells in which LRRK2 has been constitutively knocked down (Fig. 4) thus confirming the alteration of autophagy following inhibitor treatment is mediated by endogenous LRRK2 and is not a generic off-target effect. Both LRRK2-in1 and starved cells also displayed LC3 positive puncta further supporting a role for LRRK2 kinase activity as an important regulator of autophagy and suggesting that the process initiated by inhibition of LRRK2 kinase activity is biologically equivalent to autophagy induced by inhibition of mTORC1 by starvation.

**Fig. 5:**
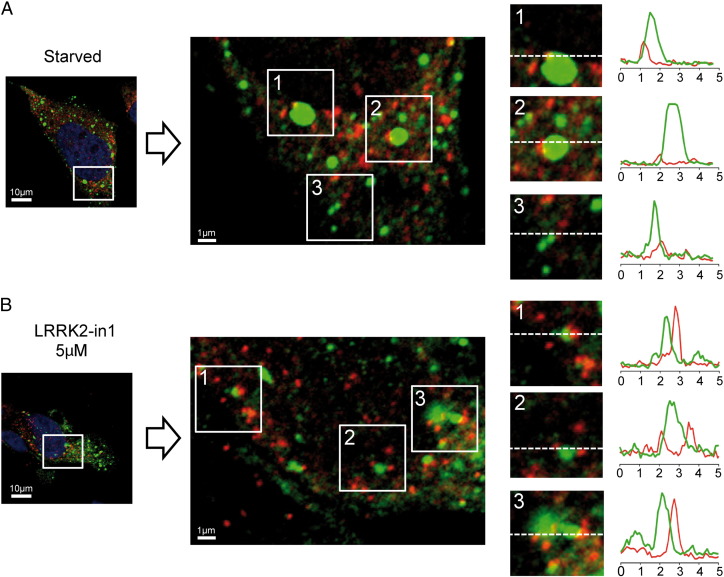
Immunocytochemical analysis for WIPI2 (red) and p62 (green) colocalization. A) starved H4 cells PCC = 0.240, Mgreen = 0.952 Mred = 0.952. B) 5 μM, LRRK2-in1 overnight treated cells PCC = 0.231, Mgreen = 0.824 Mred = 0.852. Three different spots were selected from the zoomed images and 5 μm intensity profiles were drawn as indicated by the white lines.

**Fig. 6:**
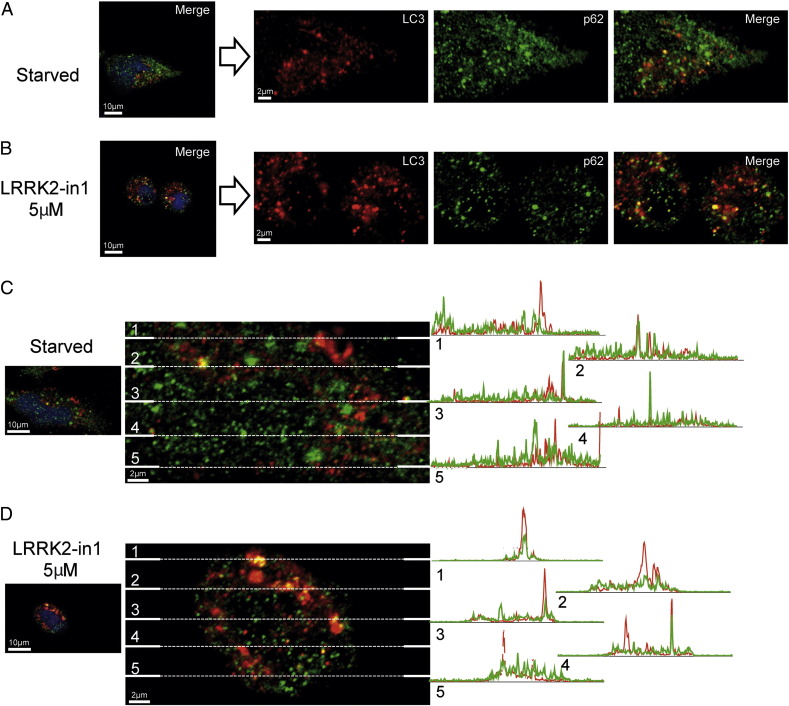
Immunocytochemical analysis for LC3 (red) and p62 (green) colocalization. A) Starved H4 cells PCC = 0.399, Mgreen = 0.830 Mred = 0.949. B) 5 μM, LRRK2-in1 overnight treated cells PCC = 0.403, Mgreen = 0.936 Mred = 0.978. C–D) images from starved or 5 μM, LRRK2-in1 overnight treated H4 cells have been crossed by 5 lines and the relative intensity profiles have been drawn to better show the partial colocalization of LC3 and p62.

To further examine the nature of the puncta produced upon inhibition of LRRK2 kinase activity, the distribution and colocalization of WIPI2, LC3 and p62, reflecting autophagosomes at different stages of maturation, were examined. Starved samples were indistinguishable from H4 cells after treatment with LRRK2-in1, demonstrating that, from a structural point of view, both treatments were able to generate the same phenotype. Many of the p62 clusters were decorated by WIPI2 staining but no proper colocalization was detected (Fig. 5 and Supplemental Movie 1). Partial colocalization between p62 and LC3 was observed (Fig. 6 and Supplemental Movie 2), as has previously been reported for endogenous levels of LC3 and p62 [39]. Importantly, there was a similar distribution of WIPI2, LC3 and p62 colocalization observed in LRRK2-in1 treated cells and in starved cells, again suggesting that the puncta induced by LRRK2 kinase inhibition are equivalent to those induced by starvation.

**Fig. 7:**
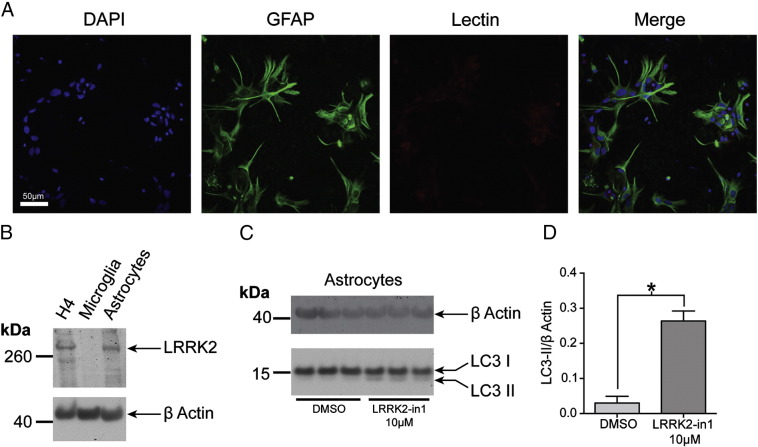
Inhibition of LRRK2 kinase activity induces autophagy in rat primary astrocytes. A) Immunocytochemical analysis of astrocytes isolated from rat brain, showing staining for DAPI, GFAP (expressed by astrocytes) and Lectin (staining for microglia). B) Astrocytes express LRRK2 at a level equivalent to H4 neuroglioma cells. C) Treatment of astrocytes with LRRK2-in1 results in an increase in LC3-II as analysed by immunoblot. D) Quantification of increase in LC3-II. The plot shows mean and SEM. * indicates significance (*p* < 0.05).

Finally, to assess how the data suggesting that inhibition of LRRK2 induces autophagy in model cell lines relates to primary cells, primary astrocytes isolated from rat brain were treated with LRRK2-in1. Astrocytes were selected as a cell type to carry out these experiments based upon previous data highlighting LRRK2 expression in these cells [30], and to march as closely as possible to the H4 cells used as the main cell model in this study (H4 cells are classified as a neuroglioma line, but are likely to have been derived from an astrocytoma [40,41]). Immunocytochemical analysis of an astrocyte specific marker (glial fibrillary acidic protein, GFAP) demonstrates successful enrichment of these cells in the *ex vivo* population, with minimal contamination with microglial cells as assessed by staining with Lectins (Fig. 7A). As previously reported [30], full length LRRK2 is expressed at an endogenous level in astrocytes, at a level comparable to that seen in H4 cells (Fig. 7B). Treatment of astrocytes with 10 μM LRRK2-in1 was not associated with detectable toxicity (data not shown), and resulted in an increase in LC3-II as previously observed for H4 cells (Figs. 7C and D) consistent with an induction of autophagy.

## 4 Discussion

Since 2004, when LRRK2 was first implicated in the pathogenesis of PD, a number of cellular processes have been linked to the function of this protein, including a putative role in the regulation of autophagy. In this study, the effect of pharmacological inhibition of LRRK2 kinase activity upon autophagy has been assessed. The results reported above document an increase in LC3-II formation and in LC3, WIPI2 and p62 positive puncta in response to treatment with an inhibitor of LRRK2 kinase activity, LRRK2-in1. Carrying out these investigations in the presence of bafilomycin, thereby examining the impact of LRRK2 kinase inhibition upon autophagic flux, suggests that the increase in LC3-II and puncta is due to an induction of autophagy — placing LRRK2 upstream of the initiation of autophagy.

A key advantage in taking a pharmacological approach is that it allows examination of endogenous LRRK2's function, removing the need to overexpress LRRK2. Due to its complicated domain structure and multiple enzymatic activities, the overexpression of LRRK2 results in the upregulation of its kinase activity, GTPase activity and its putative role as a scaffolding protein. Although it is possible that these activities are complementary, too little is known about the biology of LRRK2 to be confident that these different domains do not have divergent functions within the cell. Therefore, by using specific inhibitors against the kinase activity of LRRK2 at an endogenous level, a more reductionist approach can be taken — taking one aspect of LRRK2’s biology and examining it in isolation, in so much as this is possible. Using this approach, the data from this study confirm and support a role for LRRK2 kinase activity in the regulation of the induction of autophagy in H4 human neuroglioma cells. As outlined in Results section, these cells were chosen as a model system based upon the increasing evidence linking LRRK2 to a role in the immune system. Although the majority of the data in this study are derived from H4 cells, it should be noted that a similar effect on autophagy following the inhibition of LRRK2 kinase activity was observed in SHSY5Y cells (a neuroblastoma cell line) and, to a lesser extent, in human embryonic kidney cells, suggesting this is a consistent feature of LRRK2 kinase inhibition across a number of different cell lines (Supplemental Fig. S1). Importantly, primary astrocytes isolated from rat brain respond in a similar fashion to the H4 cells used in this study, suggesting that the results from the immortalized cell model used in this study can be at least partially extrapolated to primary cells. With regard to the nature of the vesicles created following inhibition of LRRK2 kinase activity, data from immunoblot and immunocytochemical analysis following LRRK2 kinase inhibition, carried out in parallel to the induction of autophagy by LRRK2-in1 using a starvation protocol, suggest that targeting the kinase activity of LRRK2 induces an autophagic response analogous to that observed upon inhibition of mTORC1 by starvation.

An important piece of evidence supporting this conclusion is the equivalence in the pattern of puncta produced by H4 cells in response to exposure to LRRK2-in1 and to growth under starvation condition (inhibiting mTORC1). Under both conditions, a mixed population of vesicles labeled with WIPI2, LC3 and p62 is observed. As has previously been reported [38,39], this is representative of autophagosomes at different stages in their maturation, with a proportion in each case labeled with different combinations of WIPI2, LC3 and p62. The fact that a similar pattern of colocalization is observed under both experimental conditions (and absent in the control cell population) supports the proposition that both LRRK2 kinase inhibition and starvation produce a similar autophagic response.

Despite the similarities in the autophagic response to the two experimental conditions described above, an important mechanistic finding from this study is that the induction of autophagy following LRRK2 kinase inhibition is independent of an alteration in translational downstream targets of mTORC1, S6 and 4EBP1. There are two possible explanations for this observation. First, inhibition of LRRK2 could be inducing autophagy through a specific regulation of mTORC1 activity that is independent of its role as a translational regulator. Second, LRRK2 could be regulating the induction of autophagy in an mTORC1 independent manner. Further investigations are required to distinguish between these equally intriguing possibilities, in particular directly inhibiting/knocking down mTORC1 to test whether the induction of autophagy in response to LRRK2 kinase inhibition is an mTORC1 dependent phenotype.

One difference between the autophagic response observed in starved cells *versus* that seen in LRRK2-in1 treated cells is the surprising increase in p62 levels as measured by immunoblot upon inhibition of LRRK2 kinase activity. As noted in Results section, it would be expected that the induction of autophagy would result in a decrease of in p62 levels due to its status as a degradation target for autophagy. A previous study [17] noted that manipulation of LRRK2 leads to an increase in p62 levels due to an increase in translation, and this has been replicated under the experimental conditions used in the experiments described above — inhibition of translation using the inhibitor cyclohexamide blocked the increase in p62 observed upon treatment with LRRK2-in1. While this result explains the paradoxical increase in p62 levels, it does not reveal the mechanism whereby LRRK2 inhibition results in a translational increase in p62, an observation that merits a more detailed exploration.

There are a number of caveats to the interpretation of the data in this study. First and foremost is the possibility that the cellular phenotypes observed resulted from off target effects of the LRRK2 kinase inhibitors used. To minimize the likelihood of this, two approaches have been used: first, three structurally dissimilar (including two structurally distinct) inhibitors of LRRK2 kinase function have been used, with similar cellular consequences albeit at differing concentrations. Given that LRRK2-in1, CZC-25146 and GSK 2578215A exhibit different spectrums of off target effects, the coincidence of the autophagic phenotype observed with both of these inhibitors decreases the likelihood that this is due to an impact other than a decrease in LRRK2 kinase activity. Secondly, the demonstration that LRRK2 knock down cells display a reduced response to treatment with LRRK2-in1 highlights the LRRK2 dependence of the autophagic response to LRRK2-in1 treatment. Taken together, these two sets of data strongly suggest that the induction of autophagy observed upon inhibition of LRRK2 kinase activity is a specific phenotype rather than an off target effect.

The experiments performed with LRRK2 knockdown H4 cells also raise a number of questions as to the nature of the interplay between LRRK2 and autophagy. Foremost amongst these is why, since treatment with LRRK2-in1 is a pharmacological method of reducing the activity of this protein, does the knockdown of LRRK2 not result in a similar induction of autophagy? A number of observations in the existing literature suggest why this may be the case. First, LRRK2 knockout (that is, removing LRRK2 completely from the developmental process) has a complicated, biphasic impact on steady state levels of autophagy in mice [21]. This suggests there may be a number of compensatory pathways that are engaged following the removal of LRRK2. Indeed, given the importance of autophagy to cell survival it is to be expected that multiple pathways will be involved in its regulation, resulting in a degree of redundancy in the system [42]. With reference to this, it should be noted that the H4 LRRK2 knockdown cells used in these experiments were selected over a period of several weeks to generate a pooled stable line, and so the removal of LRRK2 activity in these cells is qualitatively different to that following acute treatment of cells with LRRK2-in1. Indeed, a previous study reported that when cells are subjected to acute knockdown of LRRK2 by siRNA treatment the result is an increase in LC3-II levels, consistent with the induction of autophagy upon acute LRRK2 kinase inhibition observed in the current experiments [14].

A more complicated caveat applies to the interpretation of LRRK2 kinase activity inhibition in the context of holistic LRRK2 protein function. The aim of this study, as outlined above, was to assess the role of LRRK2 in the regulation of autophagy in a reductionist model, targeting only the kinase activity of the protein. Data from a number of studies, however, suggest that the kinase and GTPase activities of LRRK2 are intimately linked, although the exact nature of this relationship remains to be completely delineated [43–45]. This being the case, it is important to note that with current tools it is impossible to exclude a reciprocal impact on GTPase activity following the inhibition of LRRK2 kinase activity. A further complicating factor is the possibility that the two enzymatic activities may have completely separate, or even antagonistic, cellular roles [46], and this eventuality should be considered when evaluating the impact of LRRK2 kinase inhibition or gene knock down. Clarifying the roles of the different enzymatic activities of LRRK2 in its function will depend upon the development of tools to target the GTPase activity of LRRK2 [47], and is a major challenge for the field.

In summary, the data in this study provide a key insight into the mechanism whereby LRRK2 regulates autophagy, underscoring a role for the kinase activity of this protein in the control of the induction of autophagy and placing LRRK2 upstream of the initiation of this process. The experiments reported here focus on the physiological role of endogenous LRRK2, however the driving force behind LRRK2 research is the role for this protein in a number of human diseases. Although the links between kinase activity and LRRK2 PD are a matter of some discussion [48], it is clear that kinase activity does play an important, and perhaps central, role in the disease process. Given current efforts to develop LRRK2 kinase inhibitors [36], and the potential therapeutic use of these inhibitors in Parkinson's disease, it is clearly a priority to characterize the cellular impact of LRRK2 kinase inhibition, and a more detailed dissection of the mode of action of these inhibitors with regard to autophagy is an urgent need. As such, the finding that inhibiting LRRK2 kinase activity stimulates autophagy clearly has implications for the etiology of LRRK2 PD. It is also of interest that all of the diseases LRRK2 has been associated with have also had autophagy implicated in their pathogenesis [49–53]. Exploring the links between LRRK2, autophagy and human disease are, therefore, important areas for future research into this protein.

The following are the supplementary data related to this article.

Supplementary Z-stack movie 1 WIPI2-P62 colocalization
The movie 1 show the colocalization of WIPI2 (red) and P62 (green) in control, starved and inhibitor treated H4 cells. From 0 to 11 sec, 3D view of controls cells. From 12 to 38 sec, colocalization of WIPI2 and P62 in starved cells, 2D single frames. From 38 sec, to 1 min, 3D view of starved cells. From 1.01 to 1.30 min, colocalization of WIPI2 and P62 in inhibitor treated cells, 2D single frames. From 1.31 to 1.51 min, 3D view of inhibitor treated cells.

Supplementary Z-stack movie 2 LC3-P62 colocalization
The movie 2 show the colocalization of LC3 (red) and P62 (green) in control, starved and inhibitor treated H4 cells. From 0 to 20 sec, 3D view of controls cells. From 21 to 34 sec, colocalization of LC3 and P62 in starved cells, 2D single frames. From 35 to 50 sec, 3D view of starved cells. From 51 sec to 1.02 min, colocalization of LC3 and P62 in starved cells, 2D single frames (zoomed). From 1.02 to 1.18 min, 3D view of starved cells (zoomed). From 1.19 to 1.32 min, colocalization of LC3 and P62 in inhibitor treated cells, 2D single frames. From 1.33 to 1.40 min, 3D view of inhibitor treated cells. From 1.41 to 1.51 min, colocalization of LC3 and P62 in inhibitor treated cells, 2D single frames (zoomed). From 1.52 to 2.06 min, 3D view of inhibitor treated cells (zoomed).

## Funding

This study was funded by a grant from the Michael J. Fox Foundation for Parkinson's Research LRRK2 consortium to P.A.L. and R.B. C.M. is funded by the Rosetrees Trust. P.A.L. is a Parkinson's UK research fellow (grant F1002). LRRK2-in1 and CZC-25146 were provided by Dario Alessi, MRC Protein Phosphorylation Unit, University of Dundee, Dundee, Scotland. This work was supported in part by the Wellcome Trust/MRC Joint Call in Neurodegeneration award (WT089698) to the UK Parkinson's Disease Consortium (UKPDC) whose members are from the UCL Institute of Neurology, the University of Sheffield and the MRC Protein Phosphorylation Unit at the University of Dundee.
