# Supplementary figures and images for: Inhibition of LRRK2 kinase activity stimulates macroautophagy
Source: Biochim Biophys Acta. 2013 Dec;1833(12):2900–10. doi: 10.1016/j.bbamcr.2013.07.020 (PMC3898616; doi:10.1016/j.bbamcr.2013.07.020)

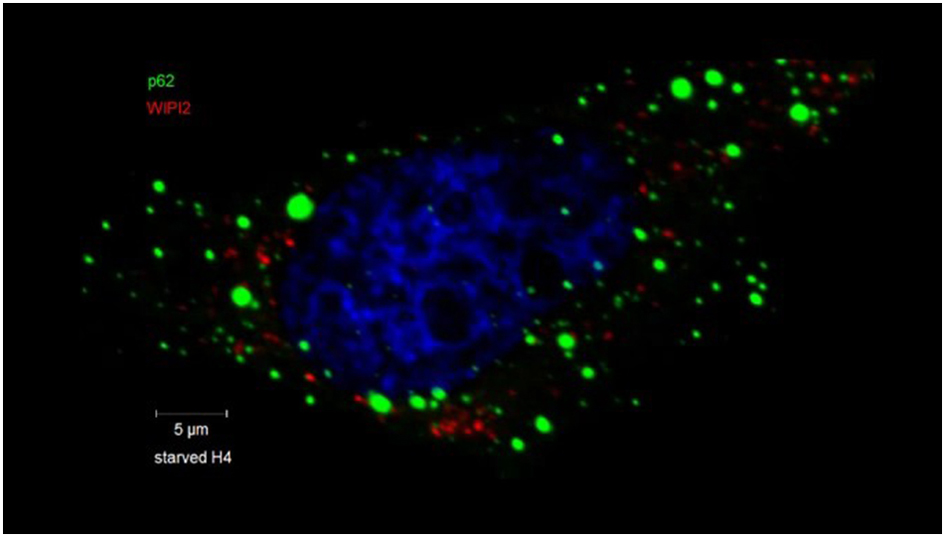

Supplement: Supplementary Z-stack movie 1 — WIPI2-P62 colocalization The movie 1 show the colocalization of WIPI2 (red) and P62 (green) in control, starved and inhibitor treated H4 cells. From 0 to 11 sec, 3D view of controls cells. From 12 to 38 sec, colocalization of WIPI2 and P62 in starved cells, 2D single frames. From 38 sec, to 1 min, 3D view of starved cells. From 1.01 to 1.30 min, colocalization of WIPI2 and P62 in inhibitor treated cells, 2D single frames. From 1.31 to 1.51 min, 3D view of inhibitor treated cells. [file mmc1.jpg]

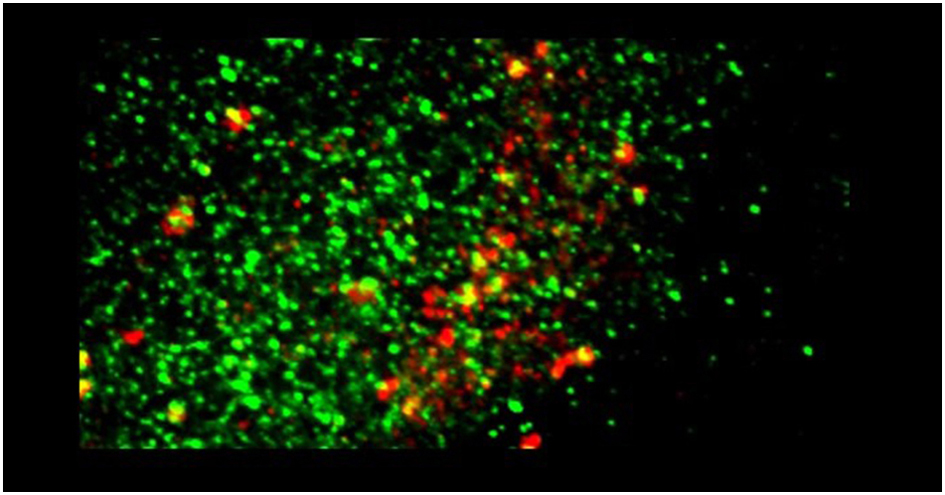

Supplement: Supplementary Z-stack movie 2 — LC3-P62 colocalization The movie 2 show the colocalization of LC3 (red) and P62 (green) in control, starved and inhibitor treated H4 cells. From 0 to 20 sec, 3D view of controls cells. From 21 to 34 sec, colocalization of LC3 and P62 in starved cells, 2D single frames. From 35 to 50 sec, 3D view of starved cells. From 51 sec to 1.02 min, colocalization of LC3 and P62 in starved cells, 2D single frames (zoomed). From 1.02 to 1.18 min, 3D view of starved cells (zoomed). From 1.19 to 1.32 min, colocalization of LC3 and P62 in inhibitor treated cells, 2D single frames. From 1.33 to 1.40 min, 3D view of inhibitor treated cells. From 1.41 to 1.51 min, colocalization of LC3 and P62 in inhibitor treated cells, 2D single frames (zoomed). From 1.52 to 2.06 min, 3D view of inhibitor treated cells (zoomed). [file mmc2.jpg]
